# Gas Concentration Prediction Based on IWOA-LSTM-CEEMDAN Residual Correction Model

**DOI:** 10.3390/s22124412

**Published:** 2022-06-10

**Authors:** Ningke Xu, Xiangqian Wang, Xiangrui Meng, Haoqian Chang

**Affiliations:** 1 State Key Laboratory of Mining Response and Disaster Prevention and Control in Deep Coal Mines, Anhui University of Science and Technology, Huainan 232000, China; nkxu999@gmail.com (N.X.); xrmeng@aust.edu.cn (X.M.); 2 School of Computer Science and Engineering, Anhui University of Science and Technology, Huainan 232000, China; 3 School of Economics and Management, Anhui University of Science and Technology, Huainan 232000, China; hqchang@aust.edu.cn

**Keywords:** coal mine safety, whale optimisation algorithm, LSTM, CEEMDAN decomposition and reconstruction, gas concentration prediction

## Abstract

In this study, to further improve the prediction accuracy of coal mine gas concentration and thereby preventing gas accidents and improving coal mine safety management, the standard whale optimisation algorithm’s (WOA) susceptibility to falling into local optima, slow convergence speed, and low prediction accuracy of the single-factor long short-term memory (LSTM) neural network residual correction model are addressed. A new IWOA-LSTM-CEEMDAN model is constructed based on the improved whale optimisation algorithm (IWOA) to improve the IWOA-LSTM one-factor residual correction model through the use of the complete ensemble empirical model decomposition with adaptive noise (CEEMDAN) method. The population diversity of the WOA is enhanced through multiple strategies and its ability to exit local optima and perform global search is improved. In addition, the optimal weight combination model for subsequence is determined by analysing the prediction error of the intrinsic mode function (IMF) of the residual sequence. The experimental results show that the prediction accuracy of the IWOA-LSTM-CEEMDAN model is higher than that of the BP neural network and the GRU, LSTM, WOA-LSTM, and IWOA-LSTM residual correction models by 47.48%, 36.48%, 30.71%, 27.38%, and 12.96%, respectively. The IWOA-LSTM-CEEMDAN model also achieves the highest prediction accuracy in multi-step prediction.

## 1. Introduction

Coal resources have long played an important role in economic development and the livelihood of people in China and will remain as the main source of energy in China for quite some time. However, coal production is a typical high-risk industry [1]. This is especially so, as the coal resources in Chinese mines continues to extend to greater depths at which the complex geological conditions under the mines result in frequent coal mine accidents. Coal mine accidents are the most frequently occurring and deadly disasters among the various types of mine accidents. Because the occurrence of coal mine gas accidents and changes in gas concentration are closely related, accurate predictions of changes in gas concentration are important for preventing gas accidents [2].

To date, many researchers have conducted extensive research on coal mine gas concentration prediction. Some results have been achieved and a number of prediction methods have been proposed, including methods based on mathematical models of gas geology as well as those based on deep learning. However, the accuracy and applicability of existing prediction methods requires further optimisation, as changes in gas concentrations in underground coal mines are influenced by a variety of complex factors, the variation trends are more complex and exhibit extreme instability and non-linearity, making it difficult to describe and predict gas transformation trends through linear relationships.

The basic principles of predicting gas concentration based on gas mathematical models are as follows: the geological rules of gas, analysis of the change rule of gas concentration, and screening of the main factors affecting the change of gas concentration. Based on these principles, according to the measured data collected in the working face and relevant geological data and considering various influencing factors comprehensively, a multi-variable mathematical model is established to predict the gas concentration in the unexploited working face of the mining area by a mathematical method. For example, based on the theory of gas geology, Lang [3] constructed a mathematical model of gas concentration from geological factors and mining factors. Liu [4] deduced the dynamic model equation of porosity and permeability from the definition of porosity, and obtained the mathematical model of coal and gas outburst by establishing the central equation of gas pressure field. Zhang [5] established a multi-variable mathematical model of gas concentration using the measured data of gas emissions in the mined area of the mine, considering various influencing factors such as the geological conditions and mining depth. However, when using the gas concentration prediction method, it is difficult to obtain the necessary input data; therefore, it is unable to realize real-time prediction. In addition, in the process of building the mathematical model, gas concentration time sequence correlation is not considered and the prediction equation requires artificial adjustment according to experience; therefore, the demand on related professional knowledge is higher. It is difficult to meet the requirements of accuracy and real-time prediction in practical production applications.

With the application of machine learning in many fields, machine learning has been applied to gas concentration prediction. For example, Lei [6] used the back propagation (BP) algorithm to construct a gas concentration prediction model based on the analysis of the nonlinear characteristics of the various factors affecting gas concentration. Peng et al. [7] used sliding Lagrange interpolation to fill in missing values and then applied the ARIMA model to predict gas concentration in real time. Cong et al. [8] used collected multidimensional gas data and environmental parameters as input features to obtain a dataset of gas data based on a sampling strategy for the construction of a feature-aware long short-term memory (LSTM) model to predict gas concentration. Dey et al. [9] predicted the gas concentration by recovering the internal features of low-dimensional data and constructing a Bi-LSTM model. Cheng [10] used a deep learning algorithm in combined with a fully connected neural network to construct an LSTM-FC gas concentration prediction model for spatiotemporal sequences using the spatiotemporal properties of gas data. Although these algorithms improve the prediction accuracy, the structure and parameters of the models can only be set artificially using empirical laws without considering the temporal correlation of the input data during the construction and training of the models.

Some intelligent optimisation algorithms have also been applied to gas concentration prediction. Liu et al. [11] constructed a hybrid GA-BP model to predict the gas concentration by exploiting the global search capability of the genetic algorithm and optimising the weights and thresholds of a BP neural network. Wang [12] et al. improved the global optimisation capability of the locust optimisation algorithm by combining multiple strategies, such as linear reduction factor reconstruction and optimal neighbourhood perturbation to optimise the relevant parameters of the LSTM model in the construction of a gas concentration prediction model. Ma [13] used the particle swarm optimization algorithm and the Adam algorithm to optimize the hyperparameters of a GRU model, so as to build a gas concentration prediction model based on PSO-Adam-GRU, which improved the prediction accuracy and robustness of the model. Fu [14] used the artificial bee colony algorithm to optimize the grid parameters of the generalized regression neural network and established a gas concentration prediction model which improved the overall generalization ability of the model. However, the above models have long hyperparameter iteration times and low convergence accuracy. They may also suffer from insufficient fitting ability when the hyperparameters are not set accurately. This will also result in insufficient prediction accuracy to meet the safety needs of underground coal mines.

To address the shortcomings of the above models, an improved whale optimisation algorithm (IWOA) is proposed in this study to optimise the LSTM residual correction model. Data processing is first performed on the gas multiparameter time series to obtain the input variables. To address the susceptibility of the whale optimisation algorithm (WOA) algorithm to local optima and slow convergence [15], the WOA algorithm is next optimised jointly through four strategies: applying a Gaussian mapping to the initial population, an elite backward learning mechanism, a reconstructed nonlinear convergence factor, and a Levy flight strategy for position updating [16]. The IWOA is then used to optimise the LSTM hyperparameters and the residual dataset of the IWOA-LSTM gas concentration prediction model is multimodally decomposed using the complete ensemble empirical model decomposition with adaptive noise (CEEMDAN) method [17]. The IWOA-LSTM model is finally used to predict the multidimensional eigenmodal components of the residual series. The optimal weights of each eigenmodal component are determined through residual assignment to construct the IWOA-LSTM-CEEMDAN residual correction model to further improve the gas concentration prediction accuracy. This model can provide strong technical support for subsequent gas risk assessment and gas accident early warning, cultivate effective safety risk analyses, enhance the reliability and efficiency of early warning, and is of great significance to improving the safety management of coal mine enterprises and reduction of the occurrence of gas accidents.

## 2. Date Source

The working face of a gas mine generates a large amount of gas during production. With reference to the sample sizes in previous gas concentration prediction studies [18,19], data were selected from 11 different measurement points at the working face of a coal mine in Guizhou from 18 March 2021 to 27 March 2021. Approximately 20,000 sets of data were obtained for short-term prediction over the next 12 h. The data attributes are summarized in Table 1.

The acquisition of high-quality underground monitoring data from coal mines has always been a concern in gas concentration prediction models. Despite the large improvements to the performance of current data collection equipment, data transmission reliability, and data storage security, a series of problems such as missing and redundant data still remain. These problems, if left unaddressed, will impact the accuracy of the subsequent gas concentration prediction models.

### 2.1. Missing Data Processing

Missing data bring serious obstacles to subsequent model training and prediction. Therefore, the Lagrange interpolation method is adopted in this paper to fill the missing data. The interpolation formula is shown as follows:

Find the nth degree polynomial such that all interpolation nodes $x_{i}$ satisfy the conditions of Formula (1).

(1) $$P_{n}\left(x_{i}\right)=y_{i}=f\left(x_{i}\right),i=1,2\ldots ,n-1\;$$

To solve $P_{n}\left(x\right)$, we construct the interpolation basis function $h_{k}\left(x\right)\left(k=0,1,\ldots ,n\right)$, which is polynomial to the nth degree and satisfies Formula (2).

(2) $$\left\{\begin{array}{c} 0,\;\;\;\;\;j\neq k\\ 1,\;\;\;\;\;j=k \end{array}\right.\;\;$$

This means that all nodes are zeros of $h_{k}\left(x\right)\;$(except $x_{k}$).

(3) $$h_{k}\left(x\right)=c\overset{n}{\underset{\begin{array}{c} j=0\\ j\neq k \end{array}}{\prod }}\left(x-x_{j}\right)\;$$

Then, determine the coefficient c in Formula (3) according to Formula (2).

(4) $$h_{k}\left(x\right)=\overset{n}{\underset{\begin{array}{c} j=0\\ j\neq k \end{array}}{\prod }}\frac{x-x_{j}}{x_{k}-x_{j}}\;$$

The solution of $P_{n}\left(x\right)$ can be obtained by using Formula (4):

(5) $$P_{n}\left(x\right)=\overset{n}{\underset{k=0}{\sum }}y_{k}h_{k}\left(x\right)=\overset{n}{\underset{k=0}{\sum }}\left(\overset{n}{\underset{\begin{array}{c} j=0\\ j\neq k \end{array}}{\prod }}\frac{x-x_{j}}{x_{k-x_{j}}}\right)y_{k}\;$$

### 2.2. PCA Data Dimensionality Reduction

With the increasing dimensionality of the data collected, the sparsity of the data become increasingly higher. Therefore, principal component analysis (PCA) was used in this study to eliminate the redundant features of the original data set. The specific steps are detailed in the following sections.

#### 2.2.1. Dataset Standardization Processing

First, assume that the data matrix composed of n-dimensional variables of m group is:

(6) $$X=\left[\begin{array}{ccc} \begin{array}{c} \begin{array}{cc} x_{11} & x_{12} \end{array}\\ \begin{array}{cc} x_{21} & x_{22} \end{array} \end{array} & \begin{array}{c} \cdots \\ \ldots \end{array} & \begin{array}{c} x_{1n}\\ x_{2n} \end{array}\\ \begin{array}{cc} \vdots & \vdots \end{array} & \ddots & \vdots \\ \begin{array}{cc} x_{m1} & x_{m2} \end{array} & \cdots & x_{mn} \end{array}\right]\;$$

where $x_{ij}(i=1,2,\ldots ,m;j=1,2,\ldots ,n)$ is the n-dimensional vector for the mth sample. $x_{ij}$ is normalized to obtain Formula (7):

(7) $$x_{ij}^{*}=\frac{x_{ij}-\bar{x_{j}}}{V_{j}}\;\left(i=1,2,\ldots ,m;j=1,2,\ldots ,n\right)\;$$

where $x_{ij}^{*}$ is the normalized value of $x_{ij}$, $\bar{x_{j}}$ is the average value of the j-dimensional variables, and $V_{j}$ is the variance of the j-dimensional variables.

#### 2.2.2. Computation of the Coefficient Matrix R

(8) $$R={\left(r_{ij}\right)}_{m*m}\;$$

where $r_{ij}=\frac{\sum_{t=1}^{n}x_{ti}x_{tj}}{n-1},\left(\;i=1,2,\ldots ,m;j=1,2,\ldots ,m,r_{ij}\right)$ is the correlation coefficient between variable $x_{i}$ and variable $x_{j}$.

#### 2.2.3. Calculation of the Cumulative Contribution Rate

(9) $$\partial_{i}=\overset{k}{\underset{i=1}{\sum }}\frac{\theta_{i}}{\sum_{i=1}^{n}\theta_{i}}\;$$

where $\theta_{i}$ is the eigenvalue corresponding to the eigenmatrix. The data after dimensionality reduction should contain the main content of the original dataset. The value of $\partial_{i}$ is generally set to more than 95%.

### 2.3. Data Normalization

In order to eliminate the dimensional influence between the gas multi-parameter time series, the data need to be normalized. The original time series is processed with the min-max standardized method, and the formula is as follows:

(10) $$y_{i}=\frac{x_{i}-min_{1\leq j\leq n}\left\{x_{j}\right\}}{max_{1\leq j\leq n}\left\{x_{j}\right\}-min_{1\leq j\leq n}\left\{x_{j}\right\}}\;$$

where $x_{1}$, $x_{2}$, …, $x_{n}$ is the original data, and the normalized data is $y_{1}$, $y_{2}$, …, $y_{n}\;$∈ [0,1] and is dimensionless.

## 3. Research Methodology

### 3.1. Whale Optimisation Algorithm

The WOA is a new intelligent population optimisation algorithm proposed by Mirjalili et al., in 2016 [20]. The algorithm is widely used because of its various advantages, which include its few adjustment parameters and simple operation. The WOA is an intelligent optimisation algorithm that simulates the predation behaviour of humpback whales. Humpback whales only hunt their prey using bubble nets. Each humpback whale position represents a feasible solution, and the principles of the algorithm are as follows:

#### 3.1.1. Surrounding the Prey

Humpback whales need to surround their prey when hunting, but the globally optimal position in the search space in practical problems is unknown. Therefore, in WOA, the initial optimal solution is set as the position of the individual whale with the highest current fitness. The other individuals will then try to approach the optimal solution and update their positions. This behaviour is described mathematically by:

(11) $$\vec{D}=\left|\vec{C}\cdot \vec{X^{*}}\left(t\right)-\vec{X}\left(t\right)\right|\;$$

(12) $$\vec{X}\left(t+1\right)=\vec{X^{*}}\left(t\right)-\vec{A}\cdot \vec{D}\;$$

where t is the current number of iterations,$\;\vec{A}$ and $\vec{C}$ are the coefficient vectors, $\vec{X^{*}}\left(t\right)$ is the current position vector of the best individual whale, and $\vec{X\left(t\right)}$ is the current position vector of the whale. $\vec{A}$ and $\vec{C}$ are given by:

(13) $$\vec{A}=2\vec{a}\cdot \vec{r_{1}}-\vec{a}\;$$

(14) $$\vec{C}=2\vec{r_{2}}\;$$

(15) $$\vec{a}=2-\frac{2t}{T_{max}}\;$$

where $\vec{r_{1}}$ and $\vec{r_{2}}$ are random vectors within [0,1] and $\vec{a}$ decreases linearly from 2 to 0 during the iteration process.

#### 3.1.2. Hunting Behaviour

Whales hunt their prey by travelling in a spiral as they contract the envelope around the prey. This behaviour of prey enclosure by the whale is realized by decreasing the value of $\vec{a}$ over the iterations. As $\vec{a}$ decreases, the value of $\vec{A}$ is taken as a random value within [−$\vec{a}$,$\vec{a}$]. When $\vec{A}$ is restricted to [−1,1], the new position vector $\vec{X}\left(t+1\right)$ will be at the initial position $\vec{X}\left(t\right)$ and the current optimal position at $\vec{X^{*}}\left(t\right)$, and the encircling behaviour of the prey is realized. The spiral behaviour of the whale as it hunts and swims towards the prey is mathematically represented as:

(16) $$\vec{X}\left(t+1\right)=\vec{D^{*}}\cdot e^{bl}\cdot \cos\left(2\pi l\right)\vec{X^{*}}\left(t\right)\;$$

where $\vec{D^{*}}=\left|\vec{X^{*}}\left(t\right)-\vec{X}\left(t\right)\right|$ denotes the distance between the whale and its prey, b is a constant used to define the logarithmic spiral motion, and l is a random number within [−1,1].

To simulate the behaviour of a whale shrinking its envelope as it travels in a spiral, it is assumed that there is a random choice between the two equally probable options of shrinking the envelope and travelling in a spiral. This manner in which the whale updates its position is mathematically represented as:

(17) $$\vec{X}\left(t+1\right)=\left\{\begin{array}{ll} \vec{X^{*}}\left(t\right)-\vec{A}\cdot \vec{D}, & if\;\;p<0.5\\ \vec{D^{*}}\cdot e^{bl}\cdot \cos\left(2\pi l\right)\vec{X^{*}}\left(t\right), & if\;\;p\gg 0.5 \end{array}\right.$$

where p is a randomly generated random number within [0,1].

#### 3.1.3. Searching for Prey

In addition to approaching a known prey, the whales will also search randomly for a new location based the positions of other whales. When $\left|\vec{A}\right|$ is set to more than 1 in the algorithm, the whales are forced to move away from the prey and find a more suitable prey, which enhances the global search capability of the algorithm. This behaviour is represented in mathematically as:

(18) $$\vec{D}=\left|\vec{C}\cdot \vec{X_{rand}}-\vec{X}\left(t\right)\right|\;$$

(19) $$\vec{X}\left(t+1\right)=\vec{X_{rand}}-\vec{A}\cdot \vec{D}\;$$

where $\vec{X_{rand}}$ represents the position vector of a randomly selected individual whale.

### 3.2. Improved Whale Optimisation Algorithm

Although WOA has achieved good results in many practical applications, the common WOA still has problems with local optimization and a low convergence accuracy. Therefore, in order to further improve the performance of the WOA algorithm, four improvement strategies are introduced, and an improved whale optimization algorithm (IWOA) is proposed.

#### 3.2.1. Gaussian Mapping

The global convergence speed and quality of the WOA optimal solution are influenced by the quality of the initial population. A population with a higher degree of diversity can improve the overall performance of the algorithm in finding the optimal solution. A Gaussian mapping is therefore used in this study to generate a more uniformly distributed initial population to improve the performance of the algorithm [21]. The Gaussian mapping is defined as:

(20) $$x_{n+1}=\left\{\begin{array}{ll} 0 & x_{n}=0\\ \frac{1}{x_{n}mod\left(1\right)} & x_{n}\neq 0 \end{array}\right.$$

The Gaussian mapping allows the states within a certain range of the search space to be maximally traversed without repetition. This ensures the diversity of the population and makes it easier for the improved algorithm to avoid local optima and therefore improving the ability of the algorithm to find the best solution.

#### 3.2.2. Elite Reverse Learning Strategy

To avoid the problem of the algorithm entering early maturity as the population diversity is increased, the elite reverse learning strategy is adopted in this study to optimise the global search ability of the algorithm. An elite group of individuals with larger fitness values among the original solution and its reverse solution are selected. The reverse solution of the elite group is solved to increase the population diversity. The best individuals from the current population and the reverse population of the elite group are then selected as the child individuals for the next iteration round [22].

Suppose $\varepsilon_{i}=\left(\varepsilon_{i,1},\varepsilon_{i,2},\ldots ,\varepsilon_{i,N}\right)\left(i=1,2,\ldots ,M\right)$ is an elite individual in the N-dimensional search space. The reverse solution $\tilde{\varepsilon_{i}}$ of this individual is defined as:

(21) $$\tilde{\varepsilon_{i}}=\left(\tilde{\varepsilon_{i,1},}\tilde{\varepsilon_{i,2}},\ldots ,\tilde{\varepsilon_{i,N}}\right)\;$$

(22) $$\tilde{\varepsilon_{i,j}}=\delta \left(\alpha b_{j}+\beta b_{j}\right)-s_{i,j}\;$$

where $\tilde{\varepsilon_{i,j}}$ represents the j-dimensional vector of the elite solution $\tilde{\varepsilon_{i}}$, δ is a random value in the interval [0,1], and $\alpha b_{j}=\max\left(\varepsilon_{i,j}\right)$ and $\beta b_{j}=\min\left(\varepsilon_{i,j}\right)$ define the dynamic boundary. The dynamic boundary not only overcomes the difficulties of preserving search experience with the fixed boundary, but also locates the elite reverse solution in the search space, which is conducive to the convergence of the algorithm. If $\tilde{\varepsilon_{i,j}}$ crosses the boundary, it needs to be reset:

(23) $$\tilde{\varepsilon_{i,j}}=rand\left(\alpha b_{j},\beta b_{j}\right),\;if\;\tilde{\;\varepsilon_{i,j}}\langle \beta b_{j}\;or\;\tilde{\;\varepsilon_{i,j}}\rangle \alpha b_{j}\;$$

where $rand\left(\alpha b_{j},\beta b_{j}\right)$ represents a random value within the interval [$\alpha b_{j},\beta b_{j}$].

#### 3.2.3. Nonlinear Adaptive Weights

Because the WOA shows a nonlinear trend in the overall optimisation process, the use of a linear inertia weight descent strategy in WOA does not provide a good representation of the actual situation during the iterative process of the algorithm. Inspired by the use of inertia weights to optimise the particle swarm optimisation (PSO) algorithm in a previous study [23], this idea is adapted for WOA by introducing a nonlinearly varying inertia weight in this study:

(24) $$\omega \left(t\right)=\omega_{max}-\left(\omega_{max}-\omega_{min}\right)*arcsin\frac{t}{t_{max}}*\frac{2}{\pi }\;$$

In the equation, $\omega_{max}$and $\omega_{min}$ represent the maximum and minimum values of ω, respectively, t is the current number of iterations, and $t_{max}$ is the maximum number of iterations. When t is small at the beginning of the iterations, the value of ω is large, and the rate of decrease of ω is slow, thereby ensuring the global search ability of the algorithm in the first iteration. As the number of iterations increases, the value of ω decreases in a nonlinear manner and the rate of decrease of ω increases rapidly, thereby ensuring good local search ability in the later stages. The use of the nonlinear varying inertia weight therefore allows the algorithm to flexibly adjust its global and local search abilities.

Substituting Equation (24) into Equations (17) and (19), the improved mathematical model for updating the position of the whale is obtained as:

(25) $$\vec{X}\left(t+1\right)=\left\{\begin{array}{ll} \omega \left(t\right)\vec{X^{*}}\left(t\right)-\vec{A}\cdot \vec{D}, & if\;\;p<0.5\\ \omega \left(t\right)\vec{D^{*}}\cdot e^{bl}\cdot \cos\left(2\pi l\right)\vec{X^{*}}\left(t\right), & if\;\;p\gg 0.5\\ \omega \left(t\right)\vec{X_{rand}}-\vec{A}\cdot \vec{D}, & if\;p<0.5\;and\;\left|\vec{A}\right|\geq 1 \end{array}\right.$$

#### 3.2.4. Levy Flight Strategy

To prevent the diversity of the WOA algorithm from decaying too quickly, which causes the algorithm to tend towards local optima, the Levy flight strategy is applied in the updating of the whale position [24]. This allows the algorithm to exit the local optima to improve its global search ability. The strategy is mathematically expressed as:

(26) $$L\left(s,\gamma ,\mu \right)=\left\{\begin{array}{l} \sqrt{\frac{\gamma }{2\pi }}\exp\left[-\frac{r}{2\left(s-\mu \right)}\right]\frac{1}{{\left(s-\mu \right)}^{3/2}}\;\;\;\;\;\;\;\;\;0<\mu <s<\infty \\ 0\;\;\;\;\;\;\;\;\;\;\;\;\;\;\;\;\;\;\;\;\;\;\;\;\;\;\;\;\;\;\;\;\;\;\;\;\;\;\;\;\;\;\;\;\;\;\;\;\;\;\;\;\;\;\;\;\;\;\;\;\;\;\;\;\;\;s\leq 0 \end{array}\right.\;\;\;\;$$

where μ is the minimum jump step and γ is a parameter greater than 0. However, the actual process for actually calculating the search path of a Levy flight is very complicated. Yang et al. [25] simplified and applied the Fourier transform to the Levy distribution function to obtain its probability density function in power form and then used the Mantegna algorithm to model the flight path as:

(27) $$\vec{X}\left(t+1\right)=\vec{X}\left(t\right)\tau L\left(s\right)\vec{X}\left(t\right)\;$$

(28) $$L\left(s\right)\sim {\left|s\right|}^{-1-\beta },\;0<\beta \leq 2\;$$

where $\vec{X}\left(t\right)$ is the current position of the individual whale, $\vec{X}\left(t+1\right)$ the updated position, and τ is the step scaling factor. s is the random step size of the Levy flight, which is expressed as:

(29) $$s=\frac{u}{{\left|v\right|}^{1/\beta }}\;$$

where both u and v obey the normal distribution, $u\sim N\left(0,\sigma_{u}^{2}\right)$, $v\sim N\left(0,\sigma_{v}^{2}\right)$. $\sigma_{u}$ and $\sigma_{v}$ are defined as:

(30) $$\left\{\begin{array}{c} \sigma_{u}=\left\{\frac{\Gamma \left(1+\beta \right)\sin\left(\pi \beta /2\right)}{\Gamma \left[\left(\left(1+\beta \right)/2\right)\right]\cdot 2^{\left(\beta -1\right)/2}}\right\}\\ \sigma_{v}=1 \end{array}\right.\;$$

where *Γ* is the standard Gamma function.

### 3.3. LSTM

**Figure 1:**
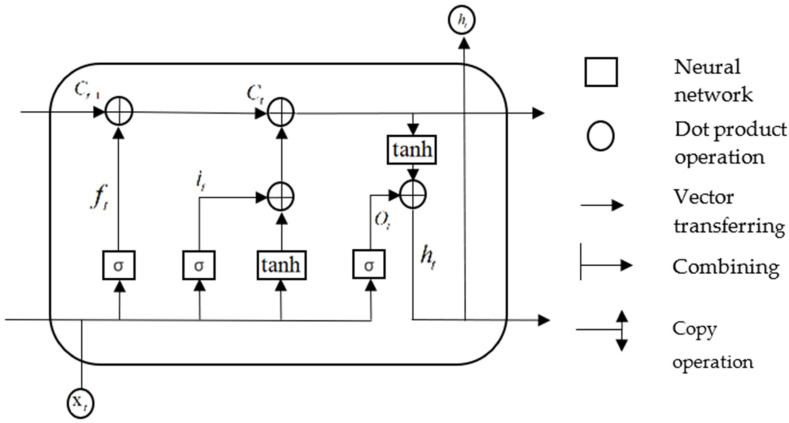
LSTM structure diagram.

Gradient disappearance and explosion may occur after multiple iterations when recurrent neural networks (RNN) are used to process time series [26]. To overcome this issue, Hochreiter et al. [27] proposed a LSTM network to improve the traditional RNN model. Compared to the hidden cells of a RNN, the hidden cells of a LSTM are more complex. The LSTM can selectively add or reduce the information content as information flows through the structure of the hidden cells. Each memory block of the LSTM consists of one or more self-connected memory cells and three gating units comprising the input, output, and forget gates. A schematic of the LSTM structure for a single cell is shown in Figure 1.

In the above structure diagram, $f_{t}$ represents the forget gate, which controls whether the hidden cellular state of the upper layer in the LSTM is filtered. $i_{t}$ represents the input gate, $C_{t-1}$ represents the cell state at the previous moment, $C_{t}$ represents the cell state at the present moment, and $O_{t}$ represents the output gate. $x_{t}$ and $h_{t}$ represent the input and output at the current moment, respectively, and σ and tanh represent the sigmoid and hyperbolic tangent functions, respectively. The forget gate, input gate, output gate, and weight matrix of the cell state are denoted by $w_{f},\;w_{i}$,$\;w_{o}$, and $w_{c}$ respectively. $b_{f},\;b_{i}$, $b_{o}$, and $b_{c}$ represent the offset vectors of the forget gate, input gate, output gate, and cell state, respectively. The calculation principles for each control gate are described below.

The value of the input gate $i_{t}$ and the candidate state value $\tilde{C}$ of the input cell at moment t are first calculated:

(31) $$i_{t}=\sigma \left(w_{i}\cdot \left[h_{t-1},x_{t}\right]+b_{i}\right)\;$$

(32) $$\tilde{C}=\tanh w_{c}\cdot \left[h_{t-1},x_{t}\right]+b_{c}\;$$

The activation value $f_{t}$ for the forget gate at moment t is then calculated:

(33) $$f_{t}=\sigma \left(w_{f}\cdot \left[h_{t-1},x_{t}\right]+b_{f}\right)\;$$

Using the values obtained in the above two steps, the cell state $C_{t}$ at moment t can be obtained:

(34) $$C_{t}=f_{t}\times C_{t-1}+i_{t}\times \tilde{C_{t}}\;$$

After obtaining the cell state update values, the output gate values can be obtained:

(35) $$O_{t}=\sigma \left(w_{o}\cdot \left[h_{t-1},x_{t}\right]+b_{o}\right)\;$$

(36) $$h_{t}=O_{t}\times \tanh\left(C_{t}\right)\;$$

Through the above calculations, the LSTM can effectively use the input function as long-term memory [28].

Overfitting is a common problem in machine learning. When the model is overfitting, the loss function of the model on the test set data is large and the prediction accuracy is low. To solve the overfitting problem, this study adds a Dropout layer to the LSTM neural network. With this, the LSTM neural network will not depend too much on specific local features so as to improve the overall generalization ability of the model and avoid the occurrence of overfitting.

### 3.4. Residual Sequence Decomposition and Reconstruction

#### 3.4.1. Residual Series Decomposition

A residual sequence $x\left(t\right)$ is constructed from the differences between the predicted and true values of the IWOA-LSTM model and used as the raw data for the subsequent multimodal decomposition.

Empirical mode decomposition (EMD) [29] is a method for dealing with the time-frequency dependence of adaptive signals by decomposing the signal into individual components that are independent of one another. Compared to the Fourier and wavelet transforms, EMD is very adaptive and powerful because it leaves the basic functions behind and only decomposes the signal based on the time-scale characteristics of the data themself. However, in practice, the time signals obtained are often anomalous, which inevitably affects the choice of the extrema and results in the envelope being a combination of anomalous and real signals. The intrinsic mode function (IMF) components filtered by this envelope are then inevitably affected by the anomalous signals and suffer from pattern overlap.

To solve the problem of mode aliasing in the EMD algorithm, Huang [30] proposed the ensemble empirical mode decomposition (EEMD) method. In this noise-assisted processing method, the uniform spectrum distribution of zero-mean noise is used in the signal analysis. When the signal is consistent with the time-frequency distribution of the white noise background throughout, the different time scales in the signal are automatically distributed to the appropriate reference scale. Because of the nature of zero-mean noise, after many repetitions the impact of the reconstruction error can be reduced by the average noise. However, the computation time of the algorithm is also greatly increased. The CEEMDAN algorithm adds a finite amount of adaptive white noise to the EMD decomposition stage to ensure that the reconstruction error is essentially zero even when the number of integration averages is small. Therefore, the CEEMDAN algorithm not only solves the modal mixing problem of the EMD algorithm, but also overcomes the shortcomings of the EEMD algorithm, which requires multiple integration averages and has low computational efficiency [31].

The detailed steps of the CEEMDAN algorithm are as follows:

1. Random white noise of a certain amplitude $n_{i}\left(t\right)$ is added to the original signal $x\left(t\right)$ to form a new signal $x_{i}\left(t\right)=x\left(t\right)+n_{i}\left(t\right),\;i=1,2,\ldots ,M$ (M is the number of averaging processes).
2. The new signal $x_{i}\left(t\right)$ is decomposed using EMD as $x_{i}\left(t\right)=\overset{n}{\underset{n=1}{\sum }}c_{i,n}\left(t\right)+r_{i}\left(t\right)$ where n is the number of EMD-decomposed IMF components, $c_{i,n}\left(t\right)$ the individual IMF components, and $r_{i}\left(t\right)$ the residual vector.
3. The overall average of the resultant n modal components gives the first eigenmodal component of the CEEMDAN decomposition: $\bar{c_{1}\left(t\right)}=\frac{1}{n}\overset{n}{\underset{n=1}{\sum }}c_{1,n}\left(t\right)$.
4. The residual when the first modal component is removed is calculated: $r_{1}\left(t\right)=x\left(t\right)-\bar{c_{1}\left(t\right)}$.
5. Using $r_{1}\left(t\right)$ as a carrier for the new signal, steps (1), (2), and (3) are repeated until the obtained residual signal cannot be further decomposed. Assuming that the number of eigenmodal components obtained at this point is k, the original signal is decomposed as $x\left(t\right)=\overset{k}{\underset{k=1}{\sum }}\bar{c_{k}\left(t\right)}+r_{k}\left(t\right)$.

**Figure 2:**
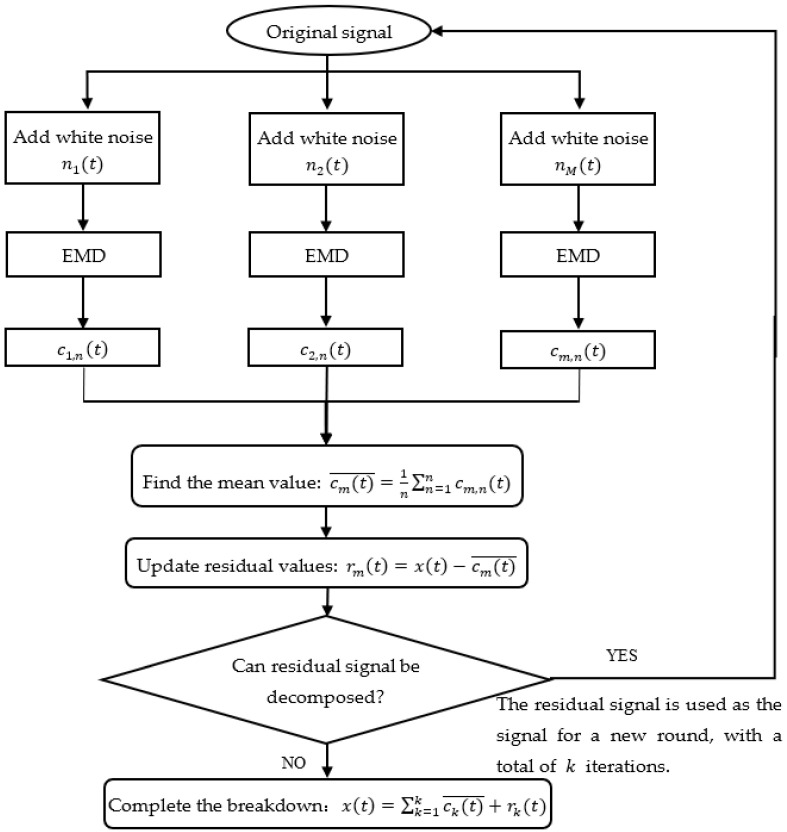
CEEMDAN decomposition flow chart.

The flow chart of the signal decomposition process in the CEEMDAN method is shown in Figure 2.

#### 3.4.2. Combination of Residual Assignments of Multidimensional Eigenmodal Components

The residual sequence $x\left(t\right)$ is decomposed using the CEEMDAN method to k subseries, consisting of the eigenmodal components. The subseries are re-divided into training and test sets and predicted using the IWOA-LSTM model. The final residual correction sequence obtained by directly adding up the prediction results of the *k* subseries will not be impacted by differences in the accuracies between different subseries. A combined residual assignment model for the k subseries is therefore used in this study. Because the range of each subseries is different, it is necessary to first normalise each subseries before residual assignment. The model is expressed as:

(37) $$\omega_{i}=\frac{\frac{1}{\varphi \left(i\right)}}{\frac{1}{\sum_{i=1}^{k}\varphi \left(i\right)}}\;$$

(38) $$\varphi \left(i\right)=\frac{1}{t}\overset{t}{\underset{\varepsilon =1}{\sum }}\left|x_{i}\left(\varepsilon \right)-\hat{x_{i}\left(\varepsilon \right)}\right|\;$$

(39) $$\hat{x}=\overset{k}{\underset{i=1}{\sum }}\omega_{i}\hat{x_{i}}\;$$

where $\omega_{i}$ represents the weight of the prediction result of the ith sub-series; $\varphi \left(i\right)$ the mean absolute error between the true and predicted values of the ith subseries; $x_{i}\left(\varepsilon \right)$ and $\hat{x_{i}\left(\varepsilon \right)}$ the true and predicted values of the εth data of the ith sub-series, respectively; $\hat{x_{i}}$ the prediction result of the ith sub-series; and $\hat{x}$ represents the data after residual correction.

### 3.5. IWOA-LSTM-CEEMDAN Residual Correction Model Construction Process

**Figure 3:**
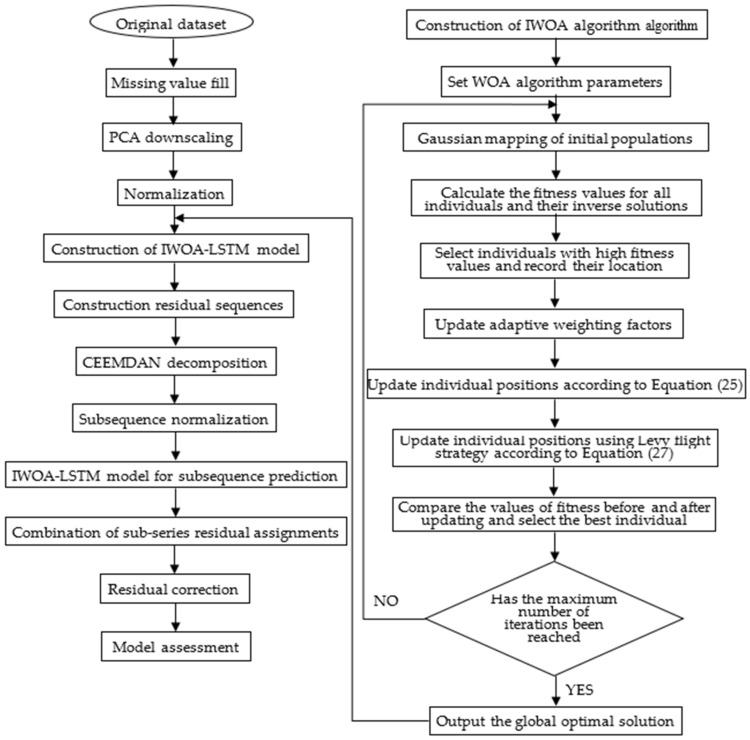
IWOA-LSTM-CEEMDAN residual correction model prediction flow chart.

A flow chart for the construction of the prediction model is shown in Figure 3. The main process is comprised of data pre-processing, improvement of the WOA, construction of the IWOA-LSTM model, decomposition and reconstruction of the residual series, and evaluation and analysis of the model’s prediction values.

1. Data pre-processing. Data pre-processing is an important process prior to data modelling because it fundamentally determines the quality of all subsequent data work and output values. The dataset for this study was sourced from a working face in a coal mine in Guizhou province. There are some missing values in the collected dataset because of network transmission issues. Therefore, the missing values in the data need to be filled, following which data downscaling and normalisation are applied on the data before suitable data are finally selected from the dataset for model training.
2. Improvement of WHO. Gaussian mapping is performed on the initialised population to produce a more uniformly distributed initial population. Elite backward learning is then performed on all the individuals in the solution space to select the optimal individual based on the fitness value. This is followed by the updating of the adaptive weight factor ω in the WOA algorithm to update the positions of the individual whales. The optimal individual is finally updated using Levy flight strategy optimisation. The fitness value of the optimised individual obtained using these four strategies is then judged to determine if the individual should be retained. This approach increases the likelihood for the algorithm to exit local optima and improve its convergence accuracy.
3. Construction of the IWOA-LSTM model. The dataset is divided into training, validation, and test sets. The IWOA-LSTM model is trained using the training set. The data from the validation set is used to monitor the model for overfitting. The data from the test set is input to the model to obtain the prediction results from the model. The differences between the true and predicted values are used as the initial data of the residual series.
4. Decomposition and reconstruction of residual sequences. Multimodal decomposition of the residual sequence is performed using the CEEMDAN method described in Section 3.4.1. The training, validation, and test sets are divided into individual eigenmodal components separately. The IWOA-LSTM model is then used to predict each eigenmodal component, and the residuals are assigned to each subsequence to obtain the predicted value of the residual-corrected sequence. This predicted value is added to the predicted value from the IWOA-LSTM model in step 3 to obtain the prediction of the final IWOA-LSTM-CEEMDAN residual-corrected model.
5. Model evaluation analysis. The forecasting ability before and after the model improvement is compared based on model evaluation indicators. The changes in the model forecasting effectiveness are analysed.

### 3.6. Evaluation Indicators

The evaluation indicators used in this study are the mean absolute error and root mean square error. Because absolute values are taken for the error values in the mean absolute error, there is no possibility of positive and negative errors cancelling one another out. The mean absolute error can therefore better reflect the actual prediction error. The existence of large errors in the predicted value will cause the value of the root mean square error to become larger; therefore, the root mean square error can effectively reflect the degree of dispersion in the error value. The explicit formulae for the errors are given by:

(40) $$MAE=\frac{1}{m}\overset{m}{\underset{i=1}{\sum }}\left|\left(y_{real}-y_{pre}\right)\right|\;$$

(41) $$RMSE=\sqrt{\frac{1}{m}\overset{m}{\underset{i=1}{\sum }}{\left(y_{real}-y_{pre}\right)}^{2}}\;$$

where m is the number of samples, $y_{real}$ is the true value, and $y_{pre}$ is the prediction result. Obviously, a smaller value of the evaluation indicator indicates a smaller error between the real and predicted values and a higher prediction accuracy of the model.

## 4. Results and Discussion

The characteristics of the dataset used in this study to verify the reliability and practicality of the proposed model are listed in Table 1, and the structure of the constructed model is shown in Figure 3. The single-step and multi-step experimental prediction results are compared with those from the BP neural network and the GRU, LSTM, WOA-LSTM, and IWOA-LSTM residual correction models, and the prediction results are analysed to show that the proposed model has higher prediction accuracy.

### 4.1. Data Dimensionality Reduction Analysis

**Figure 4:**
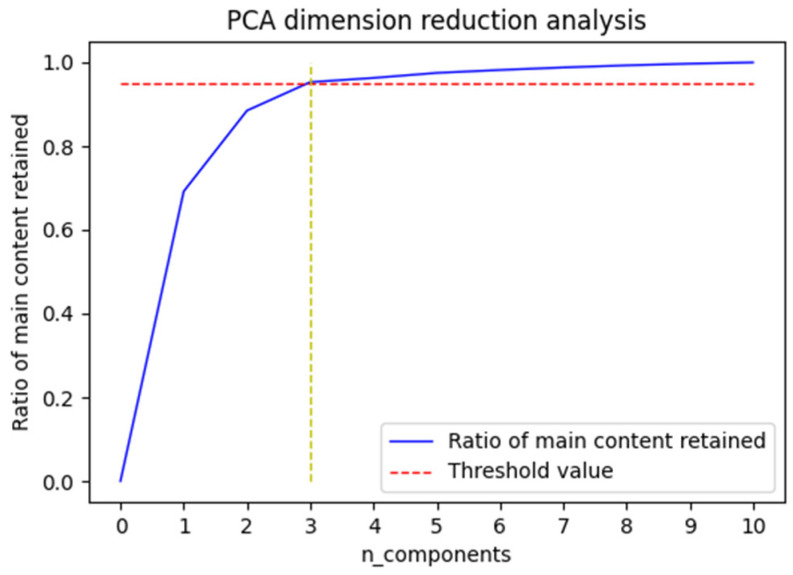
Line chart of cumulative contribution of parameters.

Missing data may exist in spatio-temporal sequence data from underground coal mines because of communication errors or network transmission delays. Because missing data can affect the prediction accuracy of the model, the missing data in the raw data were filled in using the missing data treatment method proposed in Section 2.1. After obtaining the complete dataset, PCA downscaling was performed on the dataset. Figure 4 shows the cumulative contributions of the downhole multidimensional monitoring data to the gas concentration. To cover the main components of the dataset, 98% of the main content of the original dataset needs to be retained in the data. As shown in Figure 4, the threshold value set could already be reached when the original data was downscaled from multiple dimensions to three dimensions.

To verify the necessity and validity of dimensionality reduction, the original dataset and the dataset after PCA dimensionality reduction were used as the input data for the BP, GRU, LightGBM, and LSTM models. The mean absolute errors of the prediction results from the different models are compared in Table 2.

The results of the above experiment show that the prediction accuracy of each model was significantly improved after dimensionality reduction was applied on the input dataset. The necessity and effectiveness of data dimensionality reduction were therefore verified.

### 4.2. Analysis of IWOA Algorithm

To evaluate the performance of the proposed IWOA algorithm, the 10 typical benchmark test functions shown in Table 3 were used for simulation experiments in this study [32].

**Figure 5:**
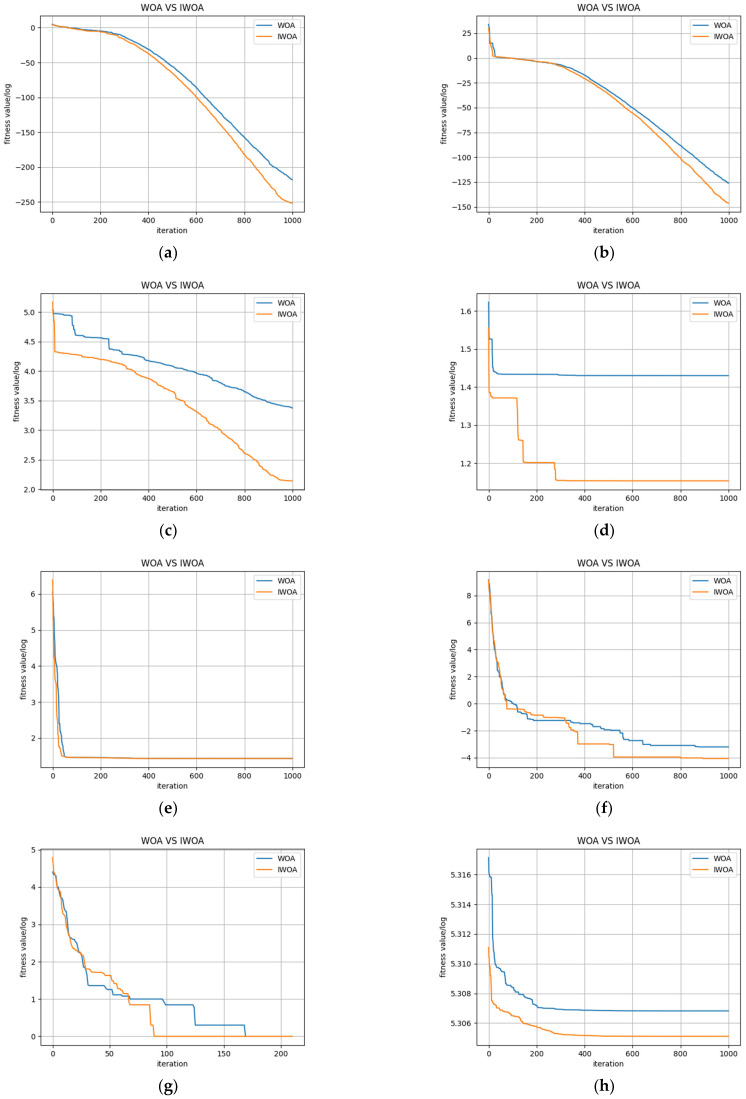

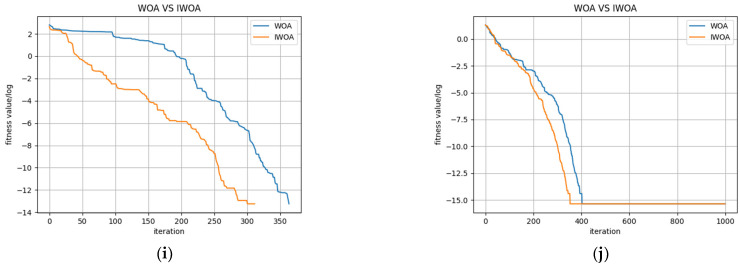
Convergence curves of each test function. (**a**) Comparison of the two algorithms on f1; (**b**) Comparison of the two algorithms on f2; (**c**) Comparison of the two algorithms on f3; (**d**) Comparison of the two algorithms on f4; (**e**) Comparison of the two algorithms on f5; (**f**) Comparison of the two algorithms on f6; (**g**) Comparison of the two algorithms on f7; (**h**) Comparison of the two algorithms on f8; (**i**) Comparison of the two algorithms on f9; (**j**) Comparison of the two algorithms on f10.

To verify the validity and effectiveness of the multi-strategy improvement of the WHO, simulations were carried out for each test function in which the maximum number of algorithm iterations was set to 1000 and the number of populations was set to 30. The optimal iterative convergence curves for each test function are shown in Figure 5.

The optimised values of the test functions used for this study were all 0. The horizontal coordinate of each curve in Figure 5 is the number of iterations, and the vertical coordinate the is value of the logarithm of the fitness value. A smaller value of the vertical coordinate corresponds to a higher convergence accuracy of the model. As shown in Figure 5, IWOA not only had high convergence accuracy but also a faster convergence speed throughout the entire search process for each given benchmark test set. These results show that IWOA has benefitted from the higher degree of diversity in the initial population and nonlinear adaptive weights, as well as the Levy flight strategy. These improvements allow the weights in IWOA to change adaptively with the current population and individual fitness values. Regions in the search space where optimal solutions may exist can therefore be rapidly searched for during the initial iterations while balancing global search and local exploitation capabilities in the process of finding the optimal solution. This avoids the process of ineffective iterations in WOA and effectively increases the convergence speed of the algorithm.

In this study, IWOA algorithm was used to optimize the hyperparameters of the LSTM network. The optimized hyperparameters of LSTM model were set as follows:” Hidden layers =2”, ”Number of neurons in the first layer = 115”, “Number of second layer neurons = 55”, ”Dropout = 0.2”, ”epoch = 11”, and ”batch-size = 256”.

### 4.3. CEEMDAN Decomposition and Reconstruction of Residual Sequences

**Figure 6:**
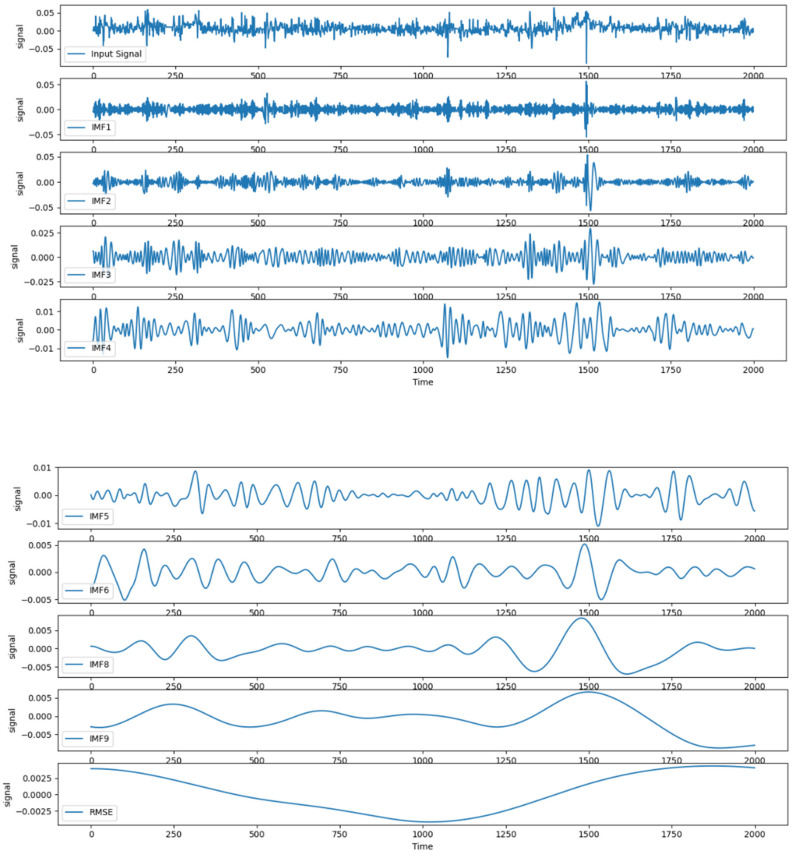
CEEMDAN decomposition of residual sequences.

Considering the non-smoothness of the residual series, the residual series was first decomposed using the CEEMDAN method. The results are shown in Figure 6.

Because the non-smoothness of the residual series results in the existence of more IMF components in the CEEMDAN decomposition, the residual assignment method was used to calculate the weights of the prediction errors of each eigenmodal component during the assignment of weights to the predicted values of each eigenmodal component. This resulted in the restructuring and merger of the subseries. The dataset of each sub-series was divided into training, validation, and test sets at a ratio of 7:2:1. The divided dataset was input into the IWOA-LSTM model for training and the data in the test set used for testing. The mean absolute errors of the true and predicted values and the weights were calculated using Equations (37) and (40), respectively, and are shown in Table 4.

Using the weights obtained from Table 4 and Equation (29), the value of the normalised reconstructed sequence could be obtained. Inverse normalisation was then performed to obtain the CEEMDAN reconstructed residual correction sequence, which was added to the predicted values from the IWOA-LSTM model to obtain the final prediction values of the IWOA-LSEM-CEEMDAN residual correction model.

### 4.4. Model Prediction Analysis and Comparison

**Figure 7:**
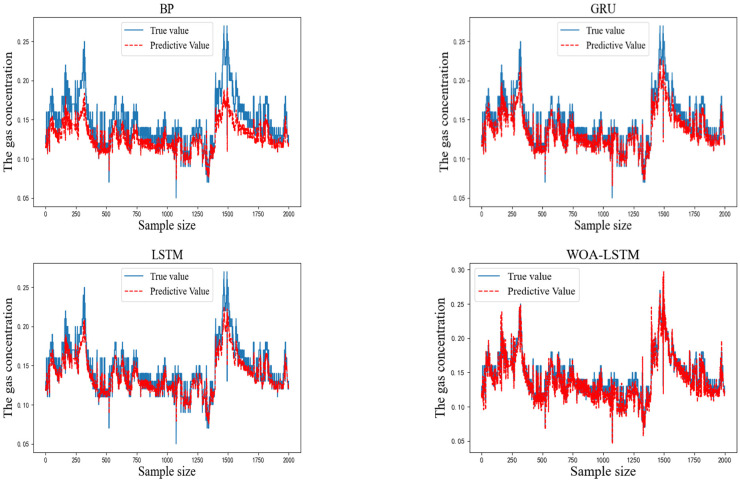

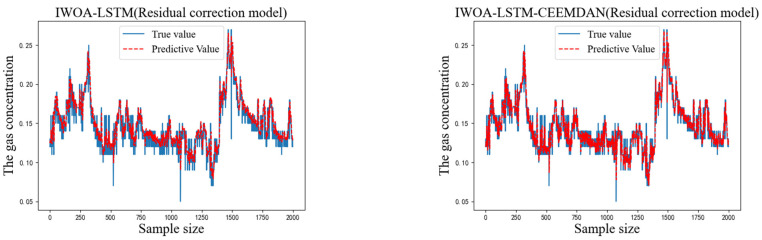
Predicted and actual results for each model.

To verify the accuracy of the proposed IWOA-LSTM-CEEMDAN residual correction model, the BP, GRU, LSTM, WOA-LSTM, and IWOA-LSTM (residual correction) models were used for comparison tests. The prediction results from the different models are shown in Figure 7.

As can be seen from the above graphs, the IWOA-LSTM-CEEMDAN residual correction model achieved a higher prediction accuracy than the traditional machine learning, WOA-LSTM, and IWOA-LSTM residual correction models. A comparison of the single-step prediction values and the multi-step prediction MAE and RMSE values (prediction step of 3) for each model is shown in Table 5.

**Figure 8:**
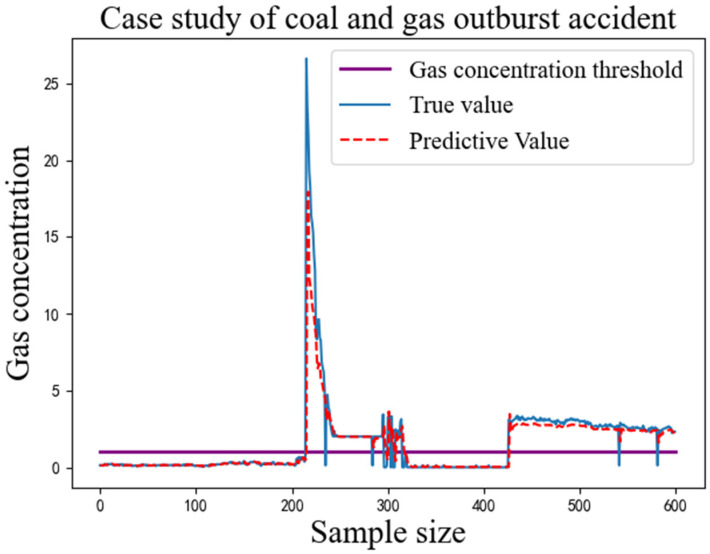
Case study of coal and gas outburst accident.

The MAE and RMSE values of each model are the average values obtained from the model trained ten times with the same parameters. The MAE and RMSE values of the IWOA-LSTM-CEEMDAN residual correction model proposed in this study are the smallest among all models for both single-step and multi-step prediction. This proves that the gas concentration prediction model proposed in this study has a higher prediction accuracy. Because the purpose of the above models is to predict gas concentrations for safe operation of gas mines, the ability of the proposed model to act as an early warning indicator for actual gas accidents was verified using the data recorded by detection equipment for the mine gas concentration on the eve of a coal and gas outburst accident in 2021. The prediction results obtained by inputting these data into the proposed model are shown in Figure 8.

As can be seen in Figure 8, the proposed model was able to replicate the gas concentration trends over a period of time before the coal and gas protrusion accident occurred. When the predicted value of the gas concentration is greater than a threshold, the manager can inform the underground operators to stop work immediately and withdraw from the mine. The gas concentration can be artificially reduced through means such as gas extraction and the spraying of water mist and the gas concentration waited to stabilise to a safe value before resuming operations.

### 4.5. Model Generalisability Analysis

**Figure 9:**
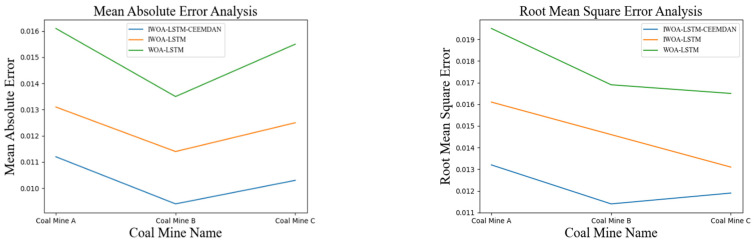
Model evaluation indicators for different coal mines.

During the selection of the study area, it was found that different regional coal mines have strong local characteristics. Coal and gas protrusion is a phenomenon involving the sudden destruction of gas-containing media in which the destroyed solids become the subject of the protrusion event. The coal structure in each coal mine is the main factor that determines the coal seam [33]. Therefore, to verify the generality of the proposed algorithm, a prediction analysis was carried out for the gas concentrations at three different coal mines comprising of the Shanxi (A), Yunnan (B), and Anhui (C) mines. The results of the comparison are shown in Figure 9.

As shown in Figure 9, the IWOA-LSTM-CEEMDAN model proposed in this study was compared with the WOA-LSTM model and the IWOA-LSTM residual correction model and validated on several coal mine datasets. The IWOA-LSTM-CEEMDAN achieved the smallest MAE and RMSE values, which further illustrates the good generalisability of the proposed prediction model.

## 5. Conclusions

To improve the accuracy of predicted gas concentrations, a multi-strategy IWOA algorithm was proposed in this study to address the shortcomings of the standard WOA algorithm. Deep learning techniques were applied for gas concentration prediction. An LSTM network model based on the IWOA algorithm was proposed, and the CEEMDAN algorithm was used to perform a multimodal decomposition and reconstruction of the prediction error of the LSTM model. The IWOA-LSTM-CEEMDAN residual correction model was then established by assigning weights to the residuals and reconstructing the residual-corrected sequences based on the prediction errors of each normalised subsequence. The following contributions were achieved in this study:

1. A multi-strategy optimisation of the WOA algorithm was proposed to improve the convergence speed and convergence accuracy of the algorithm. Through the use of benchmark test functions, the convergence accuracy and convergence speed of the improved algorithm were verified to be improved compared with those of the original model.
2. To improve the prediction accuracy of the residual correction model, a multimodal decomposition of the residual series was performed using the CEEMDAN algorithm. Each sub-series was then predicted separately, and weights were assigned to each sub-series through residual assignment. The reorganized residual correction series resulted in an obvious improvement in the prediction accuracy of the model.

A gas accident is a serious safety incident in coal mining. It endangers the safety of coal mining enterprises, causes huge economic losses and, more importantly, puts the lives of workers at risk. Therefore, it is imperative that gas concentration forecasts are undertaken. The results in this study show that it is entirely feasible to apply the IWOA-LSTM-CEEMDAN residual correction model to analyse and predict gas concentrations. These predictions can effectively reflect the future development trends of the gas concentration and provide a scientific basis for coal mining enterprises to take safety measures in advance [34]. Because of the limitations of the data in this study, the properties of the mine itself, such as the seam thickness and roof pressure, were not taken into account. In future research, more comprehensive data attributes will be collected to improve the prediction accuracy of the model, and the time step will be extended while maintaining the accuracy of the model prediction to give underground workers more time to escape in the event of a gas incident.

## Figures and Tables

**Table 1:** Data attributes of each measurement point at the working face.

Measurement Point Name	Measurement Point Description	Unit	Max Value	Min Value
MGas	Mixed methane concentration in air entry	%CH_4_	0.7	0
EGas	Methane concentration of back air in air inlet drift	%CH_4_	0.7	0
Gas1	Methane concentration in downwind side of tunnel	%CH_4_	0.79	0.16
Gas2	Methane concentration in working face of air entry	%CH_4_	0.4	0
YCO1	Concentration of carbon monoxide in downwind side of tunnel drilling	ppm	6	0
YCO2	Concentration of carbon monoxide at head of belt conveyor in air inlet lane	ppm	6	0
WS	Back air speed in air entry	m/s	1.2	0.2
FC	Dust on working face of air entry	mg/m^3^	0	0
ET	Back air temperature in air entry	°C	13.3	10.8
GD	Mixed instantaneous flow in air inlet pipeline	m^3^	19.29	0
SM	Smoke on downwind side of belt head belt driven into air entry	mg/m^3^	0	0

**Table 2:** Comparison of data prediction errors before and after dimensionality reduction for each model.

Model Name	Raw Data Prediction Error	Data Prediction Error after Dimensionality Reduction
BP model	0.0233	0.0218
GRU model	0.0198	0.0181
LightGBM model	0.0211	0.0193
LSTM model	0.0190	0.0177

**Table 3:** Functional expressions of benchmark functions.

Function Name	Function Formula	Dimensionality	Search Interval	$f_{min}$
Sphere	$f_{1}\left(x\right)=\overset{D}{\underset{i=1}{\sum }}x_{i}{}^{2}$	30	[−100,100]	0
Schwefel’Sp2.22	$f_{2}\left(x\right)=\overset{D}{\underset{i=1}{\sum }}\left|x_{i}\right|÷\prod_{i=1}^{D}\left|x_{i}\right|$	30	[−100,100]	0
Quadric	$f_{3}\left(x\right)=\overset{D}{\underset{i=1}{\sum }}{\left(\sum_{j=1}^{i}x_{j}\right)}^{2}$	30	[−100,100]	0
Schwefel’Sp2.21	$f_{4}=max_{i}\left(\left|x_{i}\right|\right),1\leq i\leq D$	30	[−50,50]	0
Rosenbrock	$f_{5}=\sum_{i=1}^{D-1}\left[100{\left(x_{i+1}-x_{i}{}^{2}\right)}^{2}+{\left(x_{i}-1\right)}^{2}\right]$	30	[−30,30]	0
Noise	$f_{6}\left(x\right)=\sum_{i=1}^{D}ix_{i}{}^{4}+random\left[0,1\right)$	100	[−10,10]	0
Step	$f_{7}\left(x\right)=\sum_{i=1}^{D}{\left(\left|x_{i}+0.5\right|\right)}^{2}$	100	[−100,100]	0
Schwefel	$f_{8}\left(x\right)=\sum_{i=1}^{D}-x_{i}\sin\left(\sqrt{\left|x_{i}\right|}\right)+418.9829\times D$	100	[−500,500]	0
Rastrigin	$f_{9}\left(x\right)=\sum_{i=1}^{D}\left[x_{i}{}^{2}-10\cos\left(2\pi x_{i}\right)÷10\right]$	100	[−10,10]	0
Ackley	$f_{10}\left(x\right)=-20\exp\left(-0.2\sqrt{\frac{1}{D}\sum_{i=1}^{D}x_{i}{}^{2}}\right)$	100	[−50,50]	0

**Table 4:** Prediction errors and weights of each subsequence.

Subsequence Name	Mean Absolute Error	Weighting
IMF1	0.00568	0.0518
IMF2	0.00625	0.0471
IMF3	0.00513	0.0573
IMF4	0.00309	0.0952
IMF5	0.00574	0.0512
IMF6	0.00272	0.1081
IMF7	0.00204	0.1442
IMF8	0.00367	0.0802
IMF9	0.00125	0.2353
RMSE	0.00124	0.2372

**Table 5:** Comparison of model evaluation indicators.

Model Name	Single-Step Prediction		Multi-Step Prediction	
	MAE	RMSE	MAE	RMSE
BP	0.01611	0.02068	0.03091	0.04468
GRU	0.01332	0.01769	0.02697	0.03524
LSTM	0.01239	0.01644	0.02778	0.03727
WOA-LSTM	0.01165	0.01513	0.02417	0.03069
IWOA-LSTM (Residual correction model)	0.00972	0.01323	0.02011	0.02796
IWOA-LSTM-CEEMDAN (Residual correction model)	0.00846	0.01239	0.01843	0.02518

## Data Availability

Data are available in a publicly accessible repository. The partial data presented in this study are openly available in [Gas concentration prediction dataset, Mendeley Data], doi:10.17632/p3n7k6hxgw.1.

## References

[1] Xu Y., Meng R.T., Zhao X. (2021). Research on a gas concentration prediction algorithm based on stacking. Sensors.

[2] Deng G. (2021). Current status and prospects of coal and gas outburst prediction and prevention technology. IOP Conf. Ser. Earth Environ. Sci..

[3] Lang X.M. (2016). Prediction of mine gas content and emission amount based on gas geology theory. Coal Technol..

[4] Liu X.Q. (2016). Mathematical model and 3D numerical simulation of coal and gas outburst. Eng. Res..

[5] Zhang Z.X. (2002). Development of mathematical model software for gas geology. Coalf. Geol. Explor..

[6] Jiang L. (2010). Construction and simulation of coal mine gas concentration prediction model based on BP neural network. Min. Saf. Environ. Prot..

[7] Wang P., Wu Y.P. (2019). Study on Lagrange-ARIMA real-time prediction model of mine gas concentration. Coal Sci. Technol..

[8] Cong Y., Zhao X., Tang K. (2022). FA-LSTM: A novel toxic gas concentration prediction model in pollutant environment. IEEE Access.

[9] Dey P., Saurabh K., Kumar C., Pandit D., Chaulya S.K., Ray S.K., Prasad G.M., Mandal S.K. (2021). t-SNE and variational auto-encoder with a bi-LSTM neural network-based model for prediction of gas concentration in a sealed-off area of underground coal mines. Soft Comput..

[10] Cheng Z.J., Ma L.Z. (2020). Spatial and temporal distribution prediction of gas concentration based on LSTM-FC. Comput. Eng. Appl..

[11] Liu Y.J., Zhao Q. (2015). Research on gas concentration prediction based on BP neural network optimized by Genetic algorithm. Min. Saf. Environ. Prot..

[12] Wang Y.H., Wang S.Y. (2021). Research on multi-parameter gas concentration prediction model based on improved locust algorithm and optimized long and short time memory neural network. J. Sens. Technol..

[13] Ma L. (2020). Prediction model of coal mine gas concentration based on PSO-Adam-GRU. J. Xi’an Univ. Sci. Technol..

[14] Fu H. (2017). Prediction of coal mine gas concentration based on improved ABC-GRNN model. Control Eng..

[15] Kaur G., Arora S. (2018). Chaotic whale optimization algorithm. J. Comput. Des. Eng..

[16] Jiang R., Yang M., Wang S., Chao T. (2020). An improved whale optimization algorithm with armed force program and strategic adjustment. Appl. Math. Model..

[17] Li J., Li Q. (2015). Research on medium term power load prediction based on CEEMDAN-permutation entropy and Leakage integral ESN. J. Electr. Mach. Control.

[18] Zhang T.J., Song S., Li S., Ma L., Pan S., Han L. (2019). Research on gas concentration prediction models based on LSTM multidimensional time series. Energies.

[19] Lyu P.Y., Chen N., Mao S., Li M. (2020). LSTM based encoder-decoder for short-term predictions of gas concentration using multi-sensor fusion. Process Saf. Environ. Prot..

[20] Mirjalili S., Lewis A. (2016). The whale optimization algorithm. Adv. Eng. Softw..

[21] Xu H., Zhang D.M. (2020). Whale optimization algorithm based on Gaussian mapping and keyhole imaging learning strategy. Comput. Appl. Res..

[22] Zhou X.Y., Wu Z.J. (2013). An elite reverse learning particle swarm optimization algorithm. Electron. J..

[23] Wang L., Wang X.K. (2007). A particle swarm optimization algorithm for nonlinear changing inertial weights. Comput. Eng. Appl..

[24] Ma W., Zhu X. (2022). Sparrow search algorithm based on Levy flight disturbance strategy. J. Appl. Sci..

[25] Yang X.S., Deb S. (2013). Multiobjective cuckoo search for design optimization. Comput. Oper. Res..

[26] Bengio Y., Simard P., Frasconi P. (1994). Learning long-term dependencies with gradient descent is difficult. IEEE Trans. Neural Netw..

[27] Hochreiter S., Schmidhuber J. (1997). Long short-term memory. Neural Comput..

[28] Shu X., Zhang L., Sun Y., Tang J. (2021). Host–parasite: Graph LSTM-in-LSTM for group activity recognition. IEEE Trans. Neural Netw. Learn. Syst..

[29] Lei Y.J., Lin J., He Z., Zuo M.J. (2013). A review on empirical mode decomposition in fault diagnosis of rotating machinery. Mech. Syst. Signal Process..

[30] Huang N.E., Shen Z., Long S.R. (1999). A new view of nonlinear water waves: The Hilbert spectrum. Annu. Rev. Fluid Mech..

[31] Hassan A.R., Bhuiyan M.I.H. (2016). Computer-aided sleep staging using Complete Ensemble Empirical Mode Decomposition with Adaptive Noise and bootstrap aggregating. Biomed. Signal Process. Control.

[32] Long W., Cai S.H. (2017). Improved whale optimization algorithm for solving large scale optimization problems. Syst. Eng. Theory Pract..

[33] Zhang Z.M., Zhang Y.G. (2005). Gas geological analysis of superlarge coal and gas outburst in Daping Coal Mine. J. Coal.

[34] Wang S.Q. (2018). Gas Time Series Prediction and Anomaly Detection Based on Deep Learning.

